# Clinical significance of mucinous component in colorectal adenocarcinoma: a propensity score-matched study

**DOI:** 10.1186/s12885-021-09031-9

**Published:** 2021-12-01

**Authors:** Chuanwang Yan, Hui Yang, Lili Chen, Ran Liu, Wei Shang, Wenguang Yuan, Fei Yang, Qing Sun, Lijian Xia

**Affiliations:** 1 Department of General Surgery, Shandong Provincial Qianfoshan Hospital, Weifang Medical University, Key Laboratory of Metabolism and Gastrointestinal Tumor, the First Affiliated Hospital of Shandong First Medical University, Key Laboratory of Laparoscopic Technology, the First Affiliated Hospital of Shandong First Medical University, Shandong Medicine and Health Key Laboratory of General Surgery, Weifang, 261000 Shandong China; 2 Department of General Surgery, The First Affiliated Hospital of Shandong First Medical University & Shandong Provincial Qianfoshan Hospital, Key Laboratory of Metabolism and Gastrointestinal Tumor, the First Affiliated Hospital of Shandong First Medical University, Key Laboratory of Laparoscopic Technology, the First Affiliated Hospital of Shandong First Medical University, Shandong Medicine and Health Key Laboratory of General Surgery, Jinan, 250000 Shandong China; 3 Department of Pathology, Jinan Central Hospital Affiliated to Shandong First Medical University, Jinan, 250000 Shandong China; 4 Department of Pathology, The First Affiliated Hospital of Shandong First Medical University & Shandong Provincial Qianfoshan Hospital, Shandong Medicine and Health Key Laboratory of Clinical Pathology, Shandong Lung Cancer Institute, Shandong Institute of Nephrology, Jinan, China

**Keywords:** Colorectal cancer, Adenocarcinoma, Mucinous component, Survival prognosis

## Abstract

**Background:**

This study aims to investigate the clinical significance and prognostic value of mucinous component (MC) in colorectal adenocarcinoma (AC).

**Methods:**

Patients with colorectal AC and AC with MC (ACMC) (1–100%) underwent surgical resection between January 2007 and February 2018 were retrospectively reviewed. Propensity score matching (PSM) was performed according to a 1:1 ratio. Receiver-operating characteristic (ROC) curve was used to identify the optimal cut-off value of MC ratio for prognostic prediction. The clinicopathological features and 3-year overall survival (OS) of AC patients, mucinous adenocarcinoma (MAC) (MC > 50%) patients, and ACMC (1–50%) patients were compared before and after matching. Multivariable analysis was used for analyzing independent risk factors related to prognosis.

**Results:**

A total of 532 patients were enrolled in this study. Patients with AC, MAC, and ACMC (1–50%) exhibited different clinicopathological features. However, their 3-year OS rates were similar (82.00% vs. 74.11% vs. 81.48%, *P* = 0.38). After matching, ROC curve determined 70% as the optimal cut-off value. And patients with ACMC > 70% had a much poorer 3-year OS compared with ACMC (1–70%) patients and AC patients (47.37% vs. 86.15% vs. 79.76%, *P* < 0.001). In addition, ACMC > 70% was revealed as a risk factor for poor survival in univariate analysis (HR = 1.643, 95%CI = 1.025–2.635, *P* = 0.039), though not an independent risk factor in multivariable analysis (HR = 1.550, 95%CI = 0.958–2.507, *P* = 0.074).

**Conclusions:**

MAC is usually diagnosed at an advanced stage. MAC has a similar survival with AC and ACMC (1–50%) patients before and after matching. Patients with ACMC > 70% exhibited a much poorer OS, and should be given more clinical attention.

## Introduction

Colorectal cancer (CRC) ranks the world’s fourth most deadly cancer with almost 900,000 deaths annually [1]. CRC has several histological types, and mucinous adenocarcinoma (MAC) comprises about 1.6–25.4% of all CRC cases [2]. According to the World Health Organization (WHO) criteria, MAC is defined as “> 50% of the lesion is composed of pools of extracellular mucin that contain malignant epithelium” [3]. However, 50% is more a cutoff value defining MAC pathologically than a clinical index indicating clinical significance and prognosis. An optimal cut-off value of mucinous proportion better defining its clinical significance is needed.

Previous studies have discovered that MAC was associated with young age, advanced tumor stage, accumulation in female patients, and distinct molecular patterns, such as microsatellite instability and activating mutations of the BRAF gene [2, 4, 5]. When analyzing clinical outcomes, the clinicopathological differences between MAC and AC are potential confounding factors. At present, findings regarding the progressive behavior and survival remain controversial in MAC [6]. Due to the lack of substantiated data, MAC specialized treatment strategy remains unclear and patients with MAC are usually treated along the lines of recommendations for adenocarcinoma (AC) of the CRC [7]. Thus, more solid evidence is needed to evaluate the significance of mucinous component (MC) in AC.

The present study aims to further evaluate the clinical significance and prognostic value of MC in AC. Slides of AC with MC (ACMC) (1–100%) were reviewed, and proportion of MC in AC was re-evaluated. Propensity-score matched (PSM) analysis was conducted to minimize bias. The optimal cut-off value of the MC proportion for prognostic prediction was analyzed. The clinicopathological features and survival of enrolled cases were also depicted before and after matching. Meanwhile, the potential risk factors for poor survival were identified.

## Materials and methods

### Study population

Records of CRC patients underwent surgical resection from January 2007 to February 2018 at the First Affiliated Hospital of Shandong First Medical University & Shandong Provincial Qianfoshan Hospital were reviewed. Final diagnosis was confirmed by pathology. Patients with a history of cancer, two or more cancers, synchronous distant metastasis, local excision, palliative surgery, and no complete clinicopathological or follow-up data were excluded. We collected the following data of each patient from clinical records: gender, age, history of smoking and alcoholism, the American Society of Anesthesiologists (ASA) class, comorbidities (hypertension, diabetes mellitus, coronary artery disease (CAD), and hepatitis), preoperative carcinoembryonic antigen (CEA), carbohydrate antigen 19–9 (CA19–9), albumin, and hemoglobin (HGB) levels, occult blood status, operative factors (operation time, perioperative blood transfusion, defunctioning stoma, and postoperative complications), and tumor factors (tumor location, differentiation, signet-ring cell component, perineural invasion (PNI), lymphovascular invasion (LVI), T stage, N stage, M stage and TNM stage). Written informed consent was signed by each patient. This study was approved by the First Affiliated Hospital of Shandong First Medical University & Shandong Provincial Qianfoshan Hospital Institutional Review Board.

### Follow-up method

Patients were followed up postoperatively every 6 months for 2 years, and then annually for 3–5 years at outpatient clinic. Physical examination, serum tumor markers, including CEA, and abdominal/chest/pelvic imaging using a CT scan were used for surveillance. Colonoscopy was performed at the 1st and 2nd year after surgery. Overall survival (OS) was defined as the period from the surgery to death from any cause.

### Pathological evaluation

For each case, the number of paraffin block for pathological evaluation was determined based on the tumor size (1 block per cm). Tumor sections from paraffin blocks were stained with hematoxylin-eosin. The ratio of MC area was separately evaluated by two pathologists, and the mean value was adopted. If the difference in estimated values was 10% or greater, the two pathologists reassessed the specimens to determine the consensus. Finally, tumors, with MC proportion ranging from 1 to 100%, were classified into 10 groups evenly with 10% ingredient per group. Classical gland-forming adenocarcinomas with variable size and configuration of the glandular structures were classified as AC. ACMC was defined as tumors with 1–100% of the lesion being composed of mucin, typically characterized by pools of extracellular mucin that contain malignant epithelium as acinar structures, strips of cells, or single cells. And those with more than 50% mucin in tumor were labelled as MAC. Signet ring cell component was defined as AC with signet ring cells, regardless of extent, which typically show displacement and molding of the nucleus.

### Statistical analysis

The data are presented as the mean and SD or as the median and range. For differences in categorical variables, chi-square analysis, Fisher exact test or Kruskal-Wallis ANOVA test was performed where appropriate. Survival was depicted with Kaplan-Meier curves and compared using log-rank tests. Univariable and multivariable survival analyses using Cox regression models were performed to identify prognostic factors. Hazard ratios (HRs) were presented with 95% confidence intervals (95%CI). Propensity-score matched (PSM) analysis was conducted to minimize bias. The 1:1 matching process was performed by using the nearest neighbor matching method, with a maximum caliper width of 0.03 times the standard deviation of the logit (propensity score). Variables adjusted included gender, age, history of smoking and alcoholism, ASA class, comorbidities, preoperative CEA, CA19–9, albumin, and HGB levels, occult blood status, operative factors and tumor factors. Receiver-operating characteristic (ROC) curve was used to identify the optimal cut-off value of MC ratio for prognostic prediction. At each ratio, the sensitivity and specificity for survival were determined and plotted, thereby generating a ROC curve. According to the (0, 1) criterion, the point on of the curve with the shortest distance to the coordinate (0, 1) was chosen as the cut-off value. Two-sided *P* ≤ 0.050 was considered statistically significant. All statistical analyses were performed using the SPSS software program (version 22.0 for Windows, IBM SPSS Statistics, IBM Corporation, Armonk, NY).

## Results

### Patient characteristics before matching

A total of 532 CRC patients were enrolled in this study. The clinicopathological features of these patients are shown in Table 1. Mean age of the patients was (64.51 ± 12.09) years, including 315 males and 217 females. Postoperative complication rate was 15.2% (81/532). As indicated in Table 1, MAC patients have a higher rate of CA19–9 ≥ 37 U/ml (*P* = 0.006), albumin< 40 g/dl (*P* = 0.006), HGB < 110 g/L (*P* = 0.007), presence of occult blood (*P* = 0.011), right-sided location (*P* < 0.001), poor differentiation (*P* < 0.001), and advanced T stage (*P* < 0.001). Other parameters were similar among the patients in the AC group, the ACMC (1–50%) group, and the MAC group (*P* > 0.05) (Table 1).

**Table 1:** Clinicopathological parameters for patients before matching

Clinicopathological parameters	Adenocarcinoma	Adenocarcinoma with mucinous component (1–50%)	Mucinous adenocarcinoma (> 50%)	*P*
Gender				0.501
Female	174	25	18	
Male	265	29	21	
Age				0.150
< 60 years	13	12	16	
≥ 60 years	306	42	23	
Smoking				0.238
No	328	44	33	
Yes	111	10	6	
Alcoholism				0.215
No	332	46	32	
Yes	107	8	7	
ASA class				0.162
II	354	38	29	
III	85	16	10	
Hypertension				0.952
No	311	39	27	
Yes	128	15	12	
Diabetes mellitus				0.691
No	381	46	32	
Yes	58	8	7	
CAD				0.386
No	398	46	34	
Yes	41	8	5	
Hepatitis				0.821
No	433	53	38	
Yes	6	1	1	
CEA				0.114
< 5 ng/ml	302	30	24	
≥ 5 ng/ml	137	24	15	
CA19–9				0.006
<37 U/ml	393	42	30	
≥ 37 U/ml	46	12	9	
Albumin				0.006
<40 g/dl	155	30	19	
≥ 40 g/dl	284	24	20	
HGB				0.007
<110 g/L	90	18	15	
≥ 110 g/L	349	36	24	
Occult blood				0.011
No	145	27	19	
Yes	294	27	20	
Operation time				0.738
< 3 h	197	25	20	
≥ 3 h	242	29	19	
Perioperative blood transfusion				0.851
No	333	41	28	
Yes	106	13	11	
Tumor location				< 0.001
Right-sided	43	16	13	
Left-sided	396	38	26	
Defunctioning stoma				0.705
No	434	53	39	
Yes	5	1	0	
Postoperative complication				0.635
Absent	374	46	31	
Present	65	8	8	
Differentiation				< 0.001
Well/Moderate	368	37	18	
Poor	71	17	21	
Signet-ring cell component				0.341
Absent	436	54	38	
Present	3	0	1	
PNI				0.735
Yes	24	3	1	
No	415	51	38	
LVI				0.551
Yes	54	8	7	
No	385	46	32	
T stage				< 0.001
1/2	138	7	3	
3/4	301	47	36	
N stage				0.240
0	224	30	15	
1/2	215	24	24	
TNM stage				0.240
I/II	224	30	15	
III	215	24	24	

*ASA* American Society of Anesthesiologists, *CAD* coronary artery disease, *CEA* carcinoembryonic antigen, *CA19–9* carbohydrate antigen 19–9, *HGB* hemoglobin, *TNM* tumor-lymph node-metastasis, *LVI* lymphovascular invasion, *PNI* perineural invasion

**Fig. 1:**
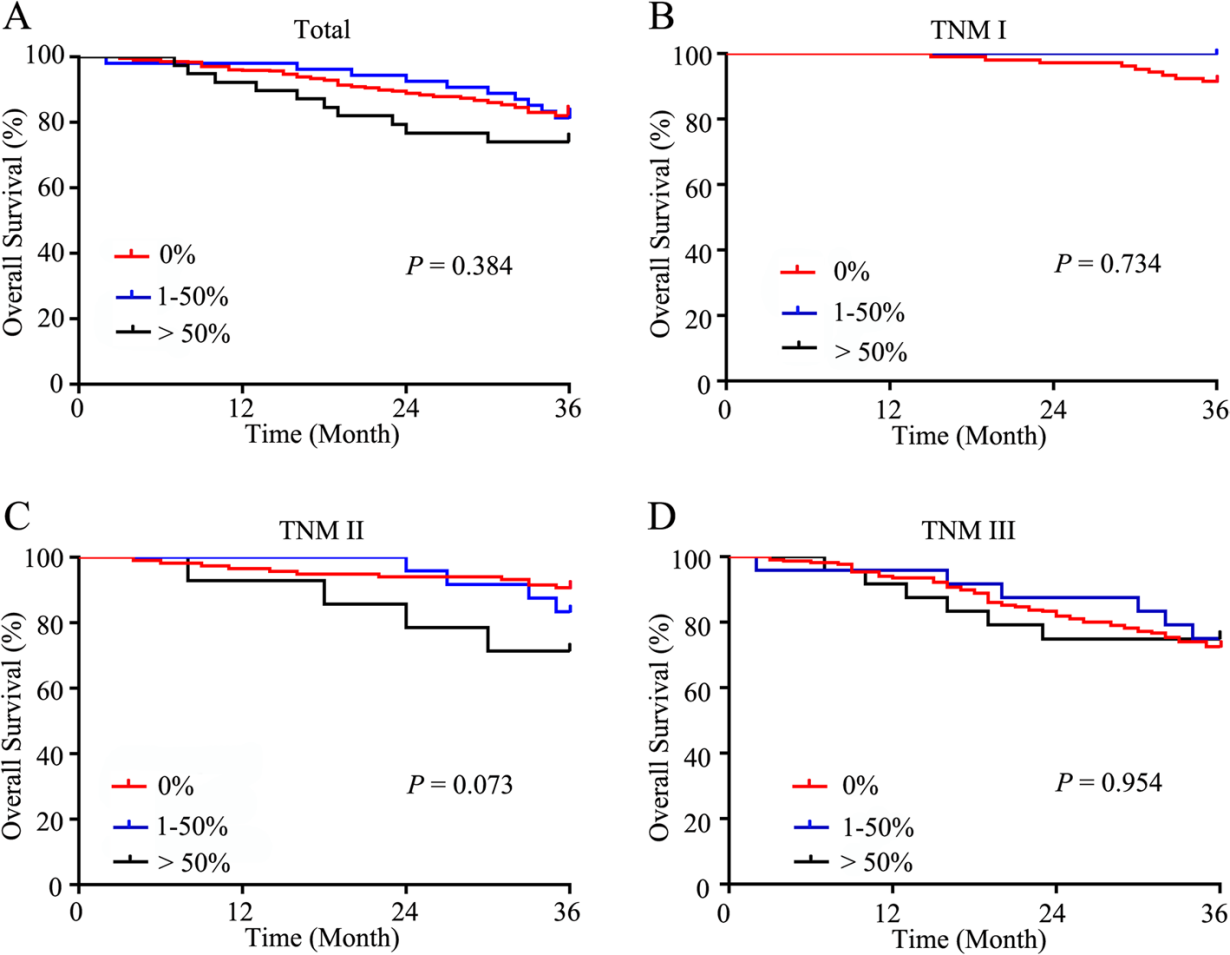
Survival of patients in the AC group, the MAC group and the ACMC (1–50%) group before matching. **A**. All involved patients. **B**. TNM stage I patients. **C**. TNM stage II patients. **D**. TNM stage III patients

The median duration of follow-up was 49 months (range, 2–170 months). The rate of patients treated with adjuvant chemotherapy was 54.32% (289/532 cases), including 236 in the AC group, 24 in the MAC group, and 29 in the ACMC (1–50%) group. The 3-year OS rates of the patients with all TNM stages, TNM stage I, II, and III were similar among the AC group (82.00, 91.51, 90.68, and 72.56%), ACMC (1–50%) group (81.48, 100, 83.33, and 75.00%), and MAC group (74.11, 100, 71.43, and 74.77%) (*P* > 0.05) (Fig. 1).

### Univariable and multivariable analyses of possible prognostic factors before matching

To identify potential risk factors for poor prognosis, univariable and multivariable analyses were conducted. The results showed that history of alcoholism (HR = 1.691, 95%CI = 1.110–2.577, *P* = 0.015), CEA ≥ 5 ng/ml (HR = 2.372, 95%CI = 1.599–3.519, *P* < 0.001), CA19–9 ≥ 37 U/ml (HR = 2.259, 95%CI = 1.406–3.629, *P* = 0.001), postoperative complication (HR = 2.312, 95%CI = 1.485–3.599, *P* < 0.001), poor differentiation (HR = 2.442, 95%CI = 1.617–3.688, *P* < 0.001), signet-ring cell component (HR = 6.603, 95%CI = 2.082–20.938, *P* = 0.001), PNI (HR = 3.712, 95%CI = 2.108–6.538, *P* < 0.001), LVI (HR = 2.709, 95%CI = 1.720–4.266, *P* < 0.001), advanced T stage (HR = 2.809, 95%CI = 1.568–5.034, *P* = 0.001), N stage (HR = 2.905, 95%CI = 1.875–4.500, *P* < 0.001), and TNM stage (HR = 3.829, 95%CI = 2.230–6.576, *P* < 0.001) were risk factors for poor OS (Table 2). When further subjecting these factors into multivariable analysis, CEA ≥ 5 ng/ml (HR = 1.830, 95%CI = 1.196–2.800, *P* = 0.005), poor differentiation (HR = 1.698, 95%CI = 1.083–2.663, *P* = 0.021), PNI (HR = 2.389, 95%CI = 1.314–4.344, *P* = 0.004), advanced N stage (HR = 1.704, 95%CI = 1.048–2.771, *P* = 0.032), and TNM stage (HR = 1.704, 95%CI = 1.048–2.771, *P* = 0.032) were identified as independent risk factors for poor OS (Table 2).

**Table 2:** Univariable and multivariable analysis for patients before matching

Parameters	Univariable analysis			Multivariable analysis		
	HR	95% CI	*P* value	HR	95% CI	*P* value
Gender Female vs. Male	1.156	0.770–1.737	0.485			
Age < 60 years vs. ≥ 60 years	1.165	0.748–1.813	0.499			
Smoking No vs. Yes	1.197	0.769–1.863	0.426			
Alcoholism No vs. Yes	1.691	1.110–2.577	0.015	1.482	0.963–2.281	0.074
ASA class II vs. III	0.982	0.601–1.605	0.943			
Hypertension No vs. Yes	0.777	0.491–1.230	0.282			
Diabetes mellitus No vs. Yes	1.145	0.661–1.984	0.628			
CAD No vs. Yes	0.668	0.310–1.440	0.303			
Hepatitis No vs. Yes	2.187	0.693–6.903	0.182			
CEA < 5 ng/ml vs. ≥ 5 ng/ml	2.372	1.599–3.519	< 0.001	1.830	1.196–2.800	0.005
CA19–9 <37 U/ml vs. ≥37 U/ml	2.259	1.406–3.629	0.001	1.327	0.793–2.222	0.281
Albumin<40 g/dl vs. ≥ 40 g/dl	0.947	0.633–1.417	0.790			
HGB <110 g/L vs. ≥110 g/L	1.475	0.874.485	0.536			
Occult blood No vs. Yes	1.013	0.671–1.530	0.950			
Operation time < 3 h vs. ≥ 3 h	0.894	0.603–1.325	0.576			
Perioperative blood transfusion No vs. Yes	1.047	0.665–1.647	0.844			
Tumor location Right-sided vs. Left-sided	0.900	0.475–1.706	0.746			
Defunctioning stoma No vs. Yes	0.915	0.128–6.561	0.930			
Postoperative complication Absent vs. Present	2.312	1.485–3.599	< 0.001	1.589	0.978–2.582	0.061
Differentiation Well/Moderate vs. Poor	2.442	1.617–3.688	< 0.001	1.698	1.083–2.663	0.021
Signet-ring cell component Absent vs. Present	6.603	2.082–20.938	0.001	1.821	0.522–6.349	0.347
Mucin No vs. Yes	1.226	0.750–2.002	0.416			
PNI Yes vs. No	3.712	2.108–6.538	< 0.001	2.389	1.314–4.344	0.004
LVI Yes vs. No	2.709	1.720–4.266	< 0.001	1.600	0.977–2.619	0.062
T stage 1/2 vs. 3/4	2.809	1.568–5.034	0.001	1.417	0.757–2.655	0.276
N stage 0 vs. 1/2	2.905	1.875–4.500	< 0.001	1.704	1.048–2.771	0.032
TNM I/II vs. III	3.829	2.230–6.576	< 0.001	1.704	1.048–2.771	0.032

*ASA* American Society of Anesthesiologists, *CAD* coronary artery disease, *CEA* carcinoembryonic antigen, *CA19–9* carbohydrate antigen 19–9, *HGB* hemoglobin, *TNM* tumor-lymph node-metastasis, *LVI* lymphovascular invasion, *PNI* perineural invasion

### PSM analysis of survival outcomes

**Fig. 2:**
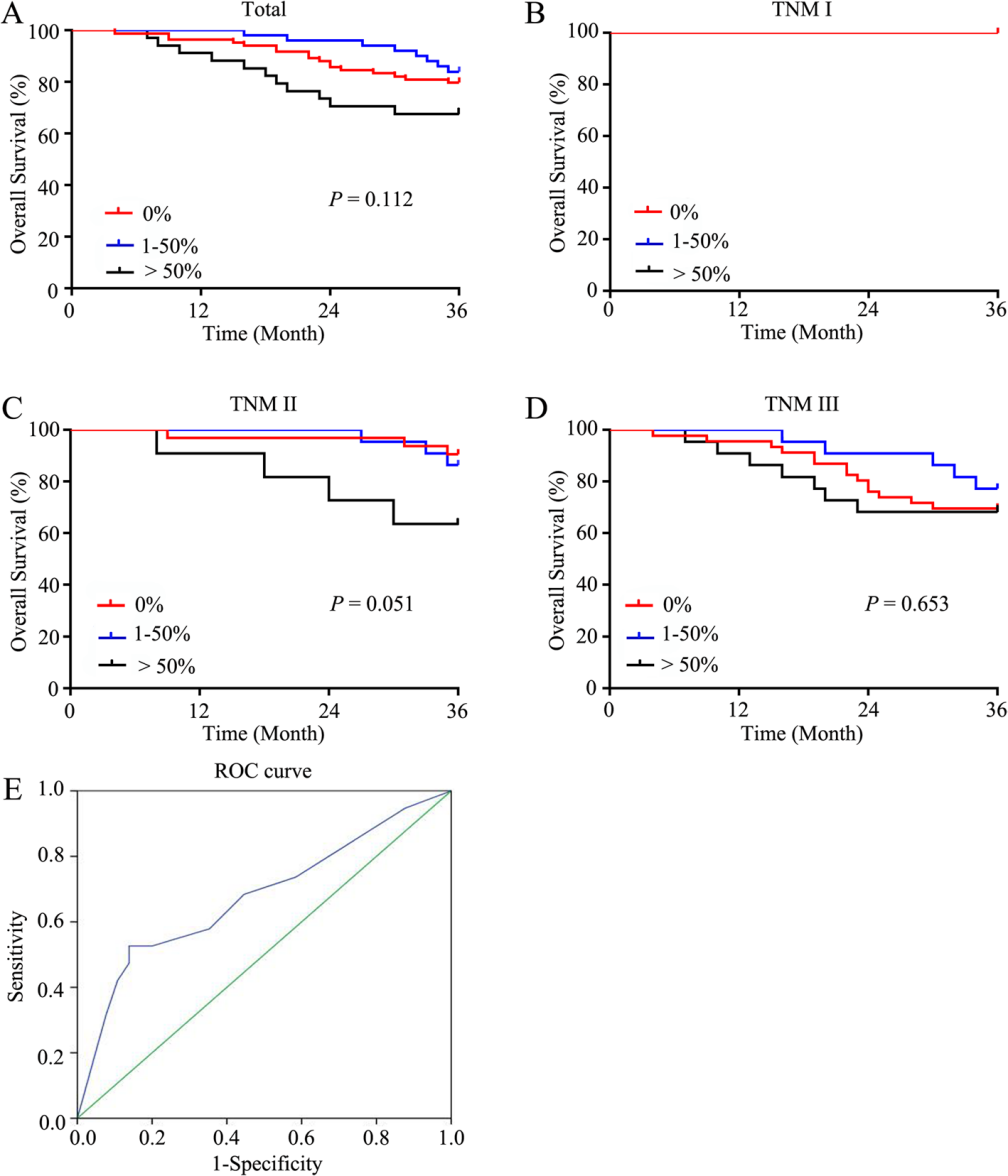
Survival of patients in the AC group, the MAC group and the ACMC (1–50%) group after matching. **A**. All involved patients. **B**. TNM stage I patients. **C**. TNM stage II patients. **D**. TNM stage III patients. **E**. ROC curve for determining the cut-off value of MC proportion for prognostic prediction

**Fig. 3:**
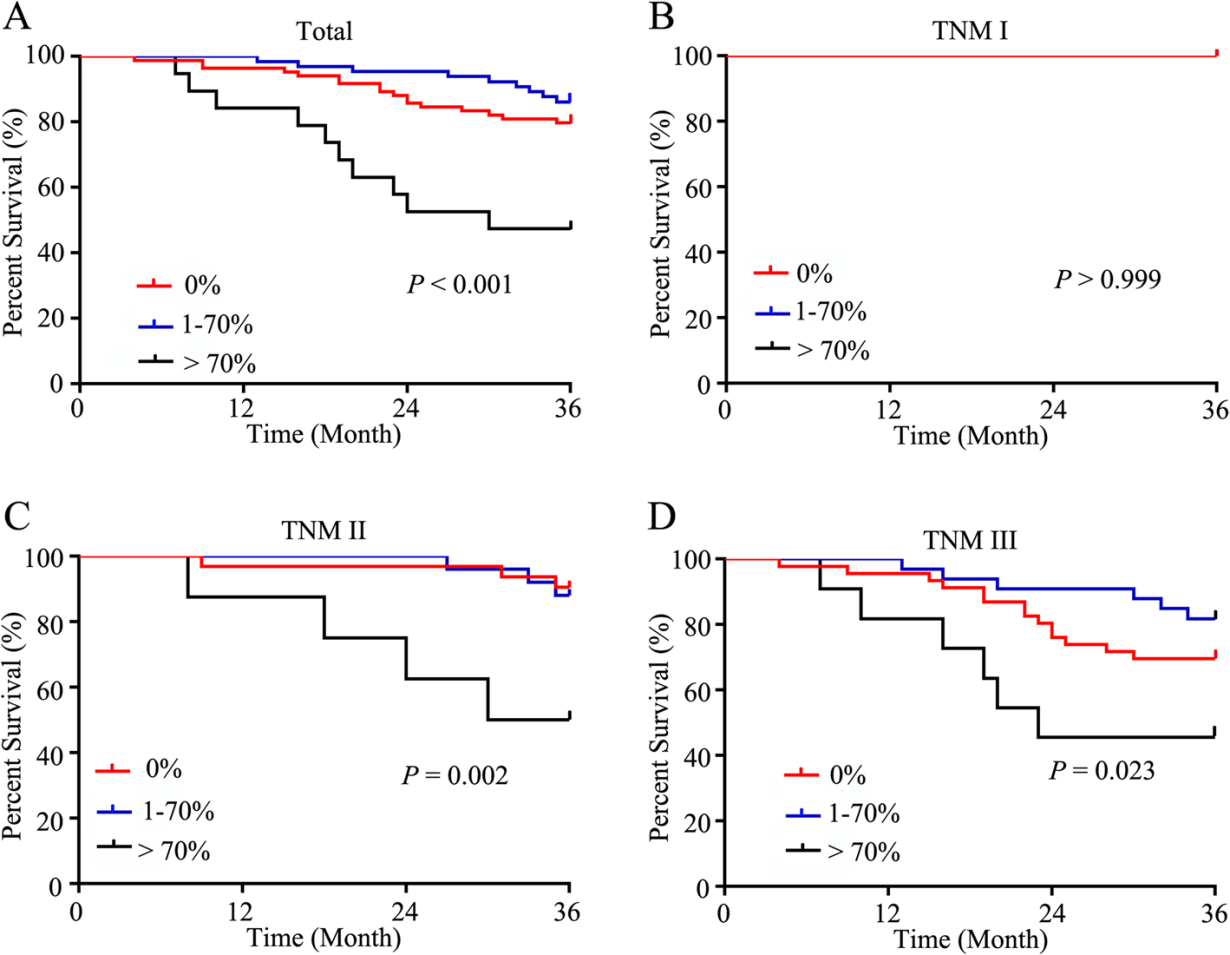
Survival of patients in the AC group, the ACMC (> 70%) group and the ACMC (1–70%) group after matching. **A**. All involved patients. **B**. TNM stage I patients. **C**. TNM stage II patients. **D**. TNM stage III patients

To account for potential imbalances, PSM analysis was conducted. As a result, 84 patients in the ACMC (1–50%) group (*n* = 50) and the MAC group (*n* = 34) were matched with 84 patients in the AC group. Mean age of the 168 patients was (65.30 ± 12.74) years. The median duration of follow-up was 49 months (range, 4–168 months). The clinicopathological features of the matched patients were similar (Table 3). The rate of patients receiving adjuvant chemotherapy was 58.33% (98/168 cases), including 49 in the AC group, 27 in the MAC group, and 22 in the ACMC (1–50%) group. The 3-year OS rates of the patients with all TNM stages, TNM stage I, II, and III were similar in the AC group (79.76, 100, 90.63, and 69.57%), ACMC (1–50%) group (84.00, 100, 86.36, and 77.27%) and the MAC group (67.65, 100, 63.64, and 68.18%) (*P* > 0.05) (Fig. 2A-D). To further to define the prognostic value of MC in CRC patients, ROC curve was adopted and 65% of mucinous area was determined as the optimal cut-off score (area under the curve = 0.677) (Fig. 2E). To increase specificity, 70% was used for the following analysis. As a result, patients with ACMC > 70% showed a much poorer survival compared with patients with ACMC (1–70%) and AC patients (47.37% vs. 86.15% vs. 79.76%, *P* < 0.001) (Fig. 3A). In addition, the prognosis was also worse in patients with ACMC > 70% in TNM stage II patients (50.00% vs. 88.00% vs. 90.63%, *P* = 0.002) and TNM stage III patients (45.46% vs. 81.82% vs. 69.57%, *P* = 0.023) (Fig. 3C-D). However, the survival was similar in TNM stage I patients (100% vs. 100%, *P* > 0.999) (Fig. 3B).

**Table 3:** Clinicopathological parameters for patients after matching

Clinicopathological parameters	Adenocarcinoma vs. Mucinous component (1–100%)	*P*	Adenocarcinoma vs. Mucinous component (1–50%)	*P*	Adenocarcinoma vs. Mucinous adenocarcinoma (> 50%)	*P*
Gender		0.537		0.418		0.051
Female	42/38		19/23		23/15	
Male	42/46		31/27		11/19	
Age		0.606		1.000		0.318
< 60 years	22/25		10/10		11/15	
≥ 60 years	62/59		40/40		23/19	
Smoking		0.694		0.349		0.709
No	67/69		23/40		31/29	
Yes	17/15		14/10		3/5	
Alcoholism		0.694		0.410		0.752
No	67/70		38/43		29/27	
Yes	17/14		12/7		5/7	
ASA class		0.860		1.000		1.000
II	62/63		36/36		26/27	
III	22/21		14/14		8/7	
Hypertension		0.733		0.829		0.582
No	61/59		34/35		26/24	
Yes	23/25		16/15		8/10	
Diabetes mellitus		0.679		0.444		1.000
No	69/71		39/42		32/31	
Yes	15/13		11/8		2/3	
CAD		0.618		0.829		1.000
No	76/74		44/43		32/31	
Yes	8/10		6/7		2/3	
Hepatitis		1.000		1.000		1.000
No	82/82		49/49		33/33	
Yes	2/2		1/1		1/1	
CEA		0.522		0.091		0.457
< 5 ng/ml	55/51		37/29		19/22	
≥ 5 ng/ml	29/33		16/21		15/12	
CA19–9		0.842		0.790		1.000
<37 U/ml	68/69		41/42		27/27	
≥ 37 U/ml	16/15		9/8		7/7	
Albumin		1.000		0.548		0.331
<40 g/dl	41/41		24/27		18/14	
≥ 40 g/dl	43/43		26/23		16/20	
HGB		0.504		0.668		0.110
<110 g/L	24/28		17/15		7/13	
≥ 110 g/L	60/56		33/35		27/21	
Occult blood		0.642		0.841		0.804
No	36/39		24/25		13/14	
Yes	48/45		26/25		21/20	
Operation time		0.642		0.689		0.215
< 3 h	46/39		25/23		11/16	
≥ 3 h	48/45		25/27		23/18	
Perioperative blood transfusion		0.452		0.640		0.109
No	68/64		37/39		31/25	
Yes	16/20		13/17		3/9	
Tumor location		0.717		1.000		0.380
Right-sided	19/21		12/12		6/9	
Left-sided	65/63		38/38		28/25	
Defunctioning stoma		1.000		1.000		1.000
No	83/83		49/49		34/34	
Yes	1/1		1/1		0/0	
Postoperative complication		1.000		0.790		0.770
Absent	68/68		41/42		27/26	
Present	16/16		9/8		7/8	
Differentiation		0.872		0.509		0.625
Well+Moderate	54/55		34/37		20/18	
Poor	30/29		16/13		14/16	
Signet-ring cell component		1.000		1.000		1.000
Absent	83/83		49/50		34/33	
Present	1/1		1/0		0/1	
PNI		1.000		1.000		1.000
No	80/80		47/47		33/33	
Yes	4/4		3/3		1/1	
LVI		0.223		1.000		0.105
No	77/72		44/44		33/28	
Yes	7/12		6/6		1/6	
T stage		0.816		0.779		1.000
1/2	11/10		8/7		3/3	
3/4	73/74		42/43		31/31	
N stage		0.757		0.230		0.324
0	38/40		22/28		16/12	
1/2	46/44		28/22		18/22	
TNM stage		0.757		0.230		0.324
I-II	38/40		22/28		16/12	
III	46/44		28/22		18/22	

*ASA* American Society of Anesthesiologists, *CAD* coronary artery disease, *CEA* carcinoembryonic antigen, *CA19–9* carbohydrate antigen 19–9, *HGB* hemoglobin, *TNM* tumor-lymph node-metastasis, *LVI* lymphovascular invasion, *PNI* perineural invasion

### Univariable and multivariable analyses of possible prognostic factors after matching

Possible prognostic factors were also analyzed by univariable and multivariable analyses after matching. As a result, MC >  70% (HR = 1.643, 95%CI = 1.025–2.635, *P* = 0.039), PNI (HR = 2.969, 95%CI = 1.049–8.400, *P* = 0.040), LVI (HR = 2.675, 95%CI = 1.218–5.878, *P* = 0.014), advanced N stage (HR = 2.555, 95%CI = 1.231–5.300, *P* = 0.012), and advanced TNM stage (HR = 2.555, 95%CI = 1.231–5.300, *P* = 0.012) were identified to be risk factors for poor OS (Table 4). Multivariable analysis found that advanced N stage (HR = 2.210, 95%CI = 1.035–4.719, *P* = 0.041) and TNM stage (HR = 2.210, 95%CI = 1.035–4.719, *P* = 0.041) were independent risk factors for poor OS (Table 4).

**Table 4:** Univariable and multivariable analysis for patients after matching

Parameters	Univariable analysis			Multivariable analysis		
	HR	95% CI	*P* value	HR	95% CI	*P* value
Gender Female vs. Male	1.301	0.671–2.524	0.436			
Age < 60 years vs. ≥ 60 years	0.626	0.317–1.235	0.177			
Smoking No vs. Yes	0.656	0.255–1.678	0.381			
Alcoholism No vs. Yes	1.076	0.471–2.456	0.862			
ASA class II vs. III	0.790	0.360–1.733	0.556			
Hypertension No vs. Yes	0.689	0.314–1.512	0.353			
Diabetes mellitus No vs. Yes	0.994	0.414–2.389	0.990			
CAD No vs. Yes	1.045	0.369–2.954	0.935			
Hepatitis No vs. Yes	1.199	0.164–8.752	0.858			
CEA < 5 ng/ml vs. ≥5 ng/ml	1.841	0.958–3.539	0.067			
CA19–9 <37 U/ml vs. ≥ 37 U/ml	1.302	0.593–2.857	0.510			
Albumin<40 g/dl vs. ≥ 40 g/dl	1.241	0.643–2.394	0.520			
HGB <110 g/L vs. ≥ 110 g/L	1.611	0.734–3.536	0.234			
Occult blood No vs. Yes	1.537	0.778–3.034	0.216			
Operation time < 3 h vs. ≥ 3 h	1.483	0.751–2.927	0.256			
Perioperative blood transfusion No vs. Yes	0.855	0.375–1.953	0.711			
Tumor location Right-sided vs. Left-sided	1.364	0.597–3.113	0.461			
Defunctioning stoma No vs. Yes	0.049	0.000–16,660.644	0.642			
Postoperative complication Absent vs. Present	1.504	0.707–3.189	0.289			
Differentiation Well/Moderate vs. Poor	1.267	0.648–2.476	0.490			
Signet-ring cell component Absent vs. Present	2.842	0.388–20.785	0.304			
Mucin No vs. Yes	1.126	0.585–2.167	0.722			
Mucin component 0% vs.1–70% vs. > 70%	1.643	1.025–2.635	0.039	1.550	0.958–2.507	0.074
PNI Yes vs. No	2.969	1.049–8.400	0.040	2.105	0.713–6.218	0.178
LVI Yes vs. No	2.675	1.218–5.878	0.014	1.687	0.721–3.944	0.228
T stage 1/2 vs. 3/4	5.531	0.758–40.375	0.092			
N stage 0 vs. 1/2	2.555	1.231–5.300	0.012	2.210	1.035–4.719	0.041
TNM I/II vs. III	2.555	1.231–5.300	0.012	2.210	1.035–4.719	0.041

*ASA* American Society of Anesthesiologists, *CAD* coronary artery disease, *CEA* carcinoembryonic antigen, *CA19–9* carbohydrate antigen 19–9, *HGB* hemoglobin, *TNM* tumor-lymph node-metastasis, *LVI* lymphovascular invasion, *PNI* perineural invasion

## Discussion

MAC has different clinicopathological features compared with AC [2, 8]. Consistently with previous reports [2, 8, 9], our data revealed that MAC was associated with higher rate of right-sided location, poor differentiation, advanced T stage before matching, which indicated that MAC is more advanced at diagnosis. In addition, our results showed that MAC patients have a higher rate of albumin< 40 g/dl, HGB < 110 g/L and presence of occult blood, these parameters were seldom analyzed in previous studies. Our data suggested that MAC patients need more nutritional support and improvement in general conditions prior to surgery.

The survival of MAC patients or ACMC (1–50%) patients has always been controversial in previous studies [2, 4, 8–24]. The retrospective nature of these studies may be an essential factor leading to the difference. Two PSM studies, minimizing confounding factors statistically, discovered that MAC was a prognostic factor in TNM stage II patients [6, 25]. This study found that the survival of patients in the AC group, the ACMC (1–50%) group, and the MAC group were similar both before matching and after matching. However, MAC exhibited a relatively low 3-year OS compared to ACMC (1–50%) and AC in TNM stage II patients after matching, though no statistical significance was detected (*P* = 0.051). However, the detailed mechanisms of MAC patients with TNM stage II exhibited poorer survival need further investigation. It has been recommended that adjuvant chemotherapy should be routinely performed for patients with stage II MAC, and special attention should be paid during follow-up because of the risk of peritoneal or local recurrence [25].

To further define the clinical significance of MC in CRC. Our study re-evaluated the MC proportion more accurately and 70% was found to be a cut-off value for predicting prognosis, which is rarely reported in previous studies. Patients with MC >  70% displayed a much poorer 3-year OS compared with patients with ACMC 1–70% and AC patients both in all patients and stage-matched (TNM stage II and stage III) patients. In addition, MC > 70% was demonstrated to be a risk factor of poor OS in univariable analysis, though not an independent risk factor to multivariable analysis. Thus, the effect of mucin on survival may be associated with its proportion in the lesion, and MC > 70% may serve as a biomarker for poor prognosis.

To better understand the cause of diverse clinical behaviors, numerous studies have focused on discovering the gene expression profiling in MAC [26–29]. Li et al. have detected that the combined mutation frequency of the two key factors of the EGFR signaling pathway, KRAS and BRAF, in the CRCs with and without MC was 95.9 and 52.1%, respectively. The desregulated EGFR pathway plays a pivotal role in the development of ACMC, irrespective of the percentage [26]. Besides, low frequency of mutations in the p53 gene or overexpression of p53 protein and loss of heterozygosity in the DCC gene have been reported [30, 31]. Genome-wide analysis found that MAC displayed 182 upregulated and 135 downregulated genes compared with AC [29]. The most upregulated genes included those involved in cellular differentiation and mucin metabolism, and altered biologic pathways included those associated with mucin substrate metabolism, amino acid metabolism, and the mitogen-activated protein kinase cascade [29]. Consistently, MUC2, which is one of the glycosylated proteins, was reported to be overexpressed in MAC [32, 33]. In addition, MAC overexpresses both TYMS and GSTP1, biomarkers indicating resistance to 5-FU and oxaliplatin [34]. These findings may partially illustrate the different phenotypes of MAC.

In conclusion, this study detected that MAC is usually diagnosed as an advanced stage. MAC patients have a similar survival with AC patients and ACMC (1–50%) patients before and after matching. Mucin accounting for more than 70% in the lesion is a more valuable cut-off score of predicting poor survival. Patients with MC > 70% should be given more clinical attention. However, data was retrospectively reviewed in this study, although PSM was conducted to adjust for known confounding factors, some degree of selection bias cannot be ruled out.

## Data Availability

The datasets used or analysed during the current study are available from the corresponding author on reasonable request.

## Abbreviations

MC: Mucinous component
AC: Colorectal adenocarcinoma
PSM: Propensity score matching
ROC: Receiver-operating characteristic
OS: Overall survival
MAC: Mucinous adenocarcinoma
HR: Hazard Ratio
CRC: Colorectal cancer
WHO: World Health Organization
ASA: The American Society of Anesthesiologists
CAD: Coronary artery disease
CEA: Carcinoembryonic antigen
HGB: Hemoglobin
PNI: Perineural invasion
LVI: Lymphovascular invasion
ACMC: With colorectal AC and AC with MC
